# Aptamer-based fluorometric determination of *Salmonella Typhimurium* using Fe_3_O_4_ magnetic separation and CdTe quantum dots

**DOI:** 10.1371/journal.pone.0218325

**Published:** 2019-06-19

**Authors:** Junan Ren, Gang Liang, Yan Man, An Li, Xinxin Jin, Qingju Liu, Ligang Pan

**Affiliations:** 1 Beijing Research Center for Agricultural Standards and Testing, Beijing Academy of Agriculture and Forestry Sciences, Beijjing, PR China; 2 Risk Assessment Lab for Agro-products (Beijing), Ministry of Agriculture, Beijing, PR China; 3 Beijing Municipal Key Laboratory of Agriculture Environment Monitoring, Beijing, PR China; Institute of Materials Science, GERMANY

## Abstract

Based on the high sensitivity and stable fluorescence of CdTe quantum dots (QDs) in conjunction with a specific DNA aptamer, the authors describe an aptamer-based fluorescence assay for the determination of *Salmonella Typhimurium*. The fluorescence detection and quantification of *S*. *Typhimurium* is based on a magnetic separation system, a combination of aptamer-coated Fe_3_O_4_ magnetic particles (Apt-MNPs) and QD-labeled ssDNA2 (complementary strand of the aptamer). Apt-MNPs are employed for the specific capture of *S*. *Typhimurium*. CdTe QD-labeled ssDNA2 was used as a signaling probe. Simply, the as-prepared CdTe QD-labeled ssDNA2 was first incubated with the Apt-MNPs to form the aptamer-ssDNA2 duplex. After the addition of *S*. *Typhimurium*, they could specifically bind the DNA aptamer, leading to cleavage of the aptamer-ssDNA2 duplex, accompanied by the release of CdTe QD-labeled DNA. Thus, an increased fluorescence signal can be achieved after magnetic removal of the Apt-MNPs. The fluorescence of CdTe QDs (λexc/em = 327/612 nm) increases linearly in the concentration range of 10 to 10^10^ cfu•mL^-1^, and the limit of detection is determined to be 1 cfu•mL^-1^. The detection process can be performed within 2 h and is successfully applied to the analysis of spiked food samples with good recoveries from 90% to 105%.

## Introduction

Bacterial infection is a major problem for human health because of their toxin system and probable antibiotic resistance [1,2]. *Salmonella Typhimurium* (*S*. *Typhimurium*) is one of the most important prevalent pathogens in humans, causing diarrhea, fever, and abdominal cramps [3,4]. This salmonellosis is mostly related to contaminated foods that mainly originate from animal sources, including poultry eggs, milk, beef, and raw food, and non-animal foods, such as fruits, vegetables, and spices [5]. Currently, *S*. *Typhimurium* is considered a key concern in many countries, such as the US, China, Europe, and Japan. Therefore, it is important to develop a method that can detect *S*. *Typhimurium* rapidly, accurately and sensitively for food quality control.

Traditional detection approaches, such as culture-based methods and color culture medium methods, have been acknowledged as routine methods for the accurate and precise detection of *S*. *Typhimurium* [6]. However, the disadvantages of plating methods for *S*. *Typhimurium* are that these methods are professional operation limited, labor intensive and time-consuming. Immunological techniques, such as enzyme-linked immunosorbent assays and immunosensors, are well established and have been used to detect *S*. *Typhimurium* for many years [7,8]. Nevertheless, the sensitivity of detection with such approaches is limited by the fact that antibodies are proteins and cannot be amplified. The conjugation, purification and preparation of antibodies are complex, time-consuming and labor-intensive. Nucleic acid sequence-based amplification methods, including PCR and real-time PCR, are also employed for the detection of *S*. *Typhimurium* and have high selectivity and sensitivity [9,10]. However, these methods require prior isolation of bacterial DNA and costly and sophisticated equipment for nucleic acid amplification and thus cannot be used routinely for real-time pathogen detection. Therefore, it is necessary to develop a low-cost, rapid, sensitive and reliable assay for *S*. *Typhimurium* detection.

Aptamers are short single-stranded nucleic acids (DNA or RNA) that are well recognized as a helpful tool for binding target substances due to their high affinity and high specificity, which are selected by systematic evolution of ligands by exponential enrichment (SELEX) [11,12]. Compared to antibodies, aptamers as chemical antibodies have more advantages, including nonimmunogenicity, small and flexible structures, high chemical production rates and stability. Moreover, aptamers can be integrated with a variety of nanomaterials, exhibiting the advantages of both aptamers and nanomaterials [13]. Quantum dots (QDs) are new and efficient fluorescent nanomaterials that are widely used in biomedical, environmental control and food security applications [14]. Compared with standard organic dyes, QDs show many advantages, including stable fluorescence, high quantum yields, narrow and symmetric emission bands, and high signal-to-noise ratios [15]. Alibolandi et al. [16] developed a sophisticated aptasensor based on aptamer-CdTe QDs to detect chloramphenicol. Bogomolova et al. [17] proposed a new aptamer-based fluorescent flow sensor with quantum dots for prolonged detection of the analyte under flow conditions.

In the present work, we developed a rapid, low-cost, sensitive and selective method for the determination of *S*. *Typhimurium* by using a magnetic separation system composed of aptamer-coated magnetic particles (Apt-MNPs) and CdTe QD-labeled complementary DNA. Apt-MNPs are employed as target captors, the CdTe QD-labeled complementary strands act as the signal generator, and the conjugates then act as the sensing probe. The sensing mechanism was characterized by TEM/HRTEM, UV-Vis spectroscopy, and fluorescence spectroscopy. The experimental observations prove the feasibility of the quantitative analysis of *S*. *Typhimurium* depending on the difference in the fluorescence signal before and after the addition of *S*. *Typhimurium*. High selectivity and sensitivity for *S*. *Typhimurium* detection are achieved, with a detection limit of 1 cfu•mL^-1^ in buffer solution. Finally, the assay was also applied to spiked food samples.

## Materials and methods

### Material and reagents

All experiments were performed with analytical reagent grade chemicals and ultrapure water (18.2 MΩ•cm) from a Milli-Q purification system (Billerica, MA). Magnetic microbeads (MBs) modified with streptavidin (1 μm, 5 mg/mL) were purchased from the BioMag Beads Company (Jiangsu, China, http://www.biomagbeads.com/). 1-Ethyl-3-(3-dimethylaminopropyl) carbodiimide hydrochloride (EDC) and N-hydroxysuccinimide (NHS) were obtained from Sigma-Aldrich (Shanghai, China, https://www.sigmaaldrich.com). Phosphate-buffered saline (PBS) was used as the washing and binding buffer and was prepared by mixing 2.04 mM Na_2_HPO_4_, 1.76 mM KH_2_PO_4_, 137 mM NaCl, and 2.68 mM KCl, which were purchased from Sinopharm Chemical Reagent Co. Ltd. (China, https://www.sinoreagent.com). Fluorescence spectra and UV-vis absorption spectra were recorded with a multimode plate reader (http://www.perkinelmer.com/). Transmission electron microscopy (TEM) images were obtained on a Hitachi HT7700 (www.hitachi.com).

*S*. *Typhimurium* (ATCC 14028), *S*. *enteritidis* (ATCC 13076), *Bacillus cereus* (ATCC 11778), *Listeria monocytogenes* (ATCC 19111), and *Staphylococcus aureus* (ATCC 25923) were obtained from the American Type Cell Collection (ATCC). *Escherichia coli* O157:H7 (CICC 21530) and *Pseudomonas aeruginosa* (CICC 10351) were obtained from the China Center of Industrial Culture Collection (CICC).

The oligonucleotides were synthesized by Shanghai Sangon Biological Science & Technology Company (Shanghai, China, http://www.sangon.com). The base sequences used in this study were as follows.

The sequence of the *S*. *Typhimurium* aptamer (ssDNA1) [18] is 5’-biotin-C_6_-TATGGCGGCGTCACCCGACGGGGACTTGACATTATGACAG-3’.

The amino-modified complementary sequence (ssDNA2) is 5’-C_6_-NH2-CTGTCATAATGTCAAGTC-3’. The aptamer was isolation from outer membrane proteins (OMPs) of *S*. *Typhimurium* using the SELEX protocol [18].

### Preparation of CdTe QDs

**Fig 1:**
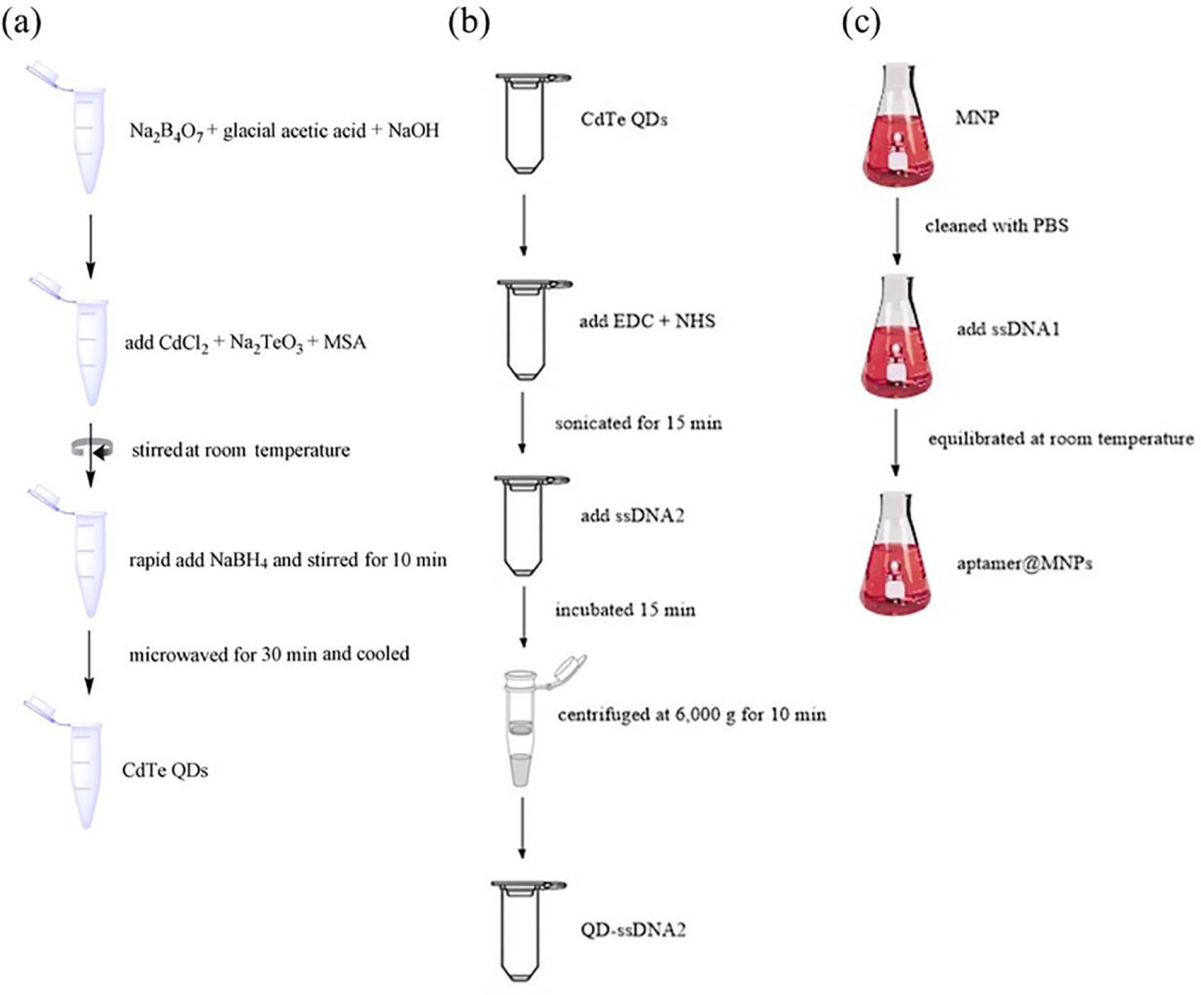
The flow chart diagram of synthesis of QDs (a), QDs-ssDNA2 (b) and aptamer@MNPs (c).

The CdTe QDs were synthesized in an aqueous solution according to a previously published method [19]. Briefly, 342 mg Na_2_B_4_O_7_ (17 mM) and 100 μL glacial acetic acid (17 mM) were mixed in 1 M NaOH. Then, 20 mg CdCl_2_ (1 mM), 4.4 mg Na_2_TeO_3_ (0.2 mM) and 45 mg mercaptosuccinic acid (MSA, 3 mM) were added to the above borate buffer solution and stirred at room temperature. After that, 46 mg of NaBH_4_ was rapidly added to the solution and stirred for 10 min. The resulting solution was microwaved for 30 min and cooled to 30°C. The prepared carboxyl-capped CdTe QDs were stored in a dark bottle at 4°C (Fig 1A).

Next, we conjugated ssDNA2 to the carboxyl-capped CdTe QDs. Then, 50 μL EDC (20 mM) and NHS (20 mM) were added to 50 μL CdTe QD solution and sonicated for 15 min to activate the carboxyl groups of CdTe QDs. Then, 25 μg ssDNA2 was added and incubated for 15 min at 4°C. After that, the mixture was centrifuged at 6000 × g for 10 min with a 50 kD Millipore filter. The samples were then redispersed in 200 μL PBS (pH 7.4) (Fig 1B).

### Fabrication of fluorescence detection assay materials

The aptamer@MNPs were prepared first. Ten microliters of 1 mg•mL^-1^ streptavidin-modified MNP solution was cleaned with PBS (pH 7.4). Then, 50 μL of 0.01 nM biotin-aptamer (ssDNA1) was added to the solution and equilibrated at room temperature for 30 min. Then, 50 μL QDs-ssDNA2 was added to the aptamer@MNPs solution and gently shaken for 15 min at 37°C to rapidly hybridize. Finally, the excess QDs-ssDNA2 in the supernatant was removed by magnetic separation. The product aptamer& QDs-ssDNA2@MNPs were redispersed in 50 μL PBS (pH 7.4) (Fig 1C).

### Detection of *S*. *Typhimurium*

The bacterial strains were inoculated and grown in Luria-Bertani broth at 37°C with gentle shaking at 180 rpm for 18–24 h until the optical density value at 600 nm (OD600) reached 0.3. Then, the mixture was centrifuged at 12,000 rpm for 5 min to remove the broth. Then, 200 μL PBS (pH 7.4) was added to the tube to dissolve the pellet. The solution was transferred to the aptamer&QDs-ssDNA2@MNPs suspension and then placed on an orbital shaker for 30 min at 37°C. After magnetic separation, the supernatant was monitored by fluorescence spectroscopy with irradiation at 612 nm. To determine the limit of detection (LOD), we employed the following formula: LOD = (3 × standard deviation)/slope, n = 10.

### Treatment of milk and water samples

To verify the validity of the assay in practical products, we purchased milk and water samples from the local market. The milk sample was centrifuged at 8500 rpm for 10 min, and the upper cream layer was removed. Then, the supernatant was filtered with a 0.45-μm filtration membrane and diluted at a 1:20 ratio with ultrapure water. Each diluted *S*. *Typhimurium* sample was spiked in the milk and water samples for detection.

## Results and discussion

### Principle of the fluorescence assay

**Fig 2:**
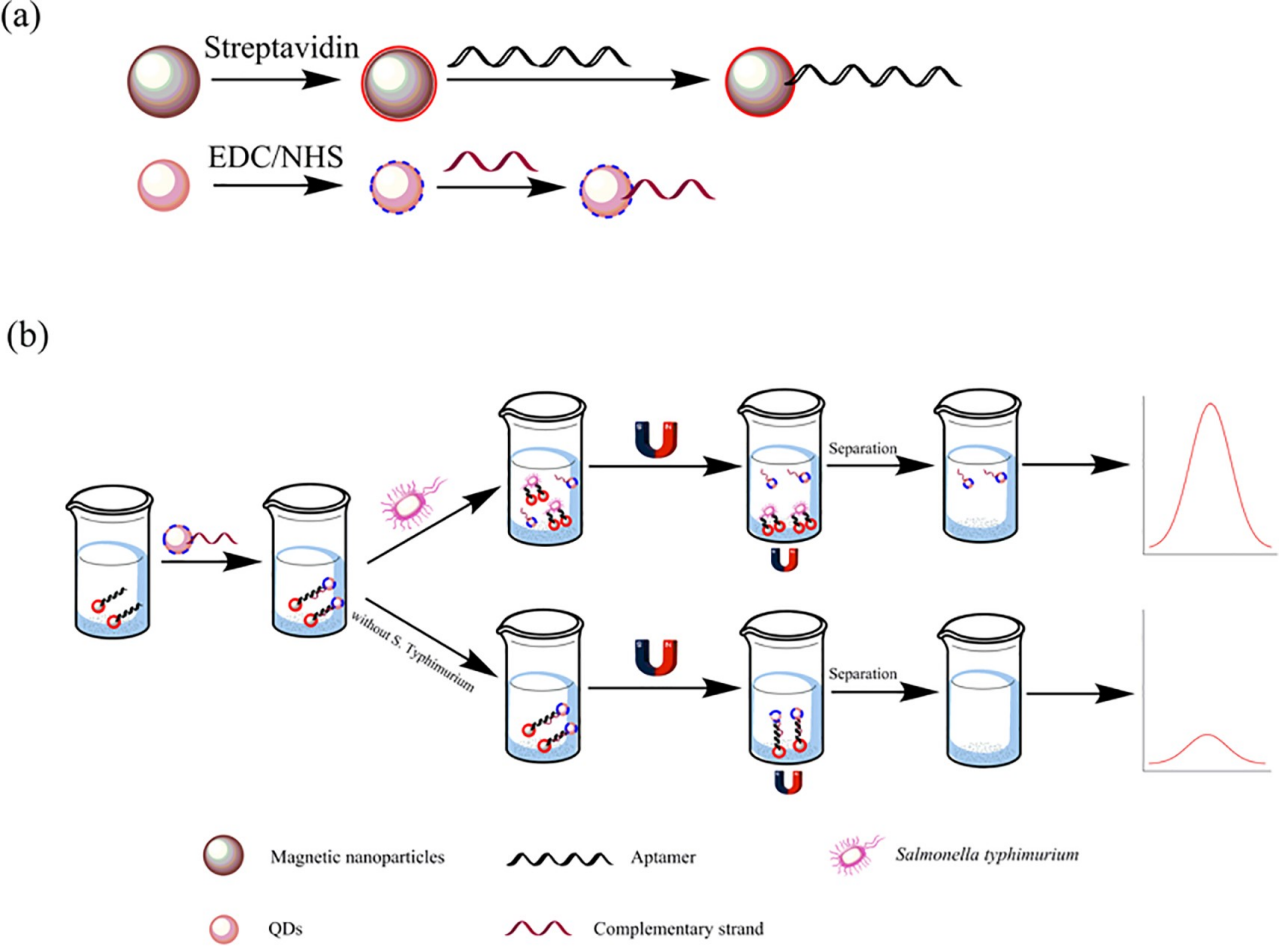
Schematic diagram of (**a**) the synthesis of streptavidin magnetic nanoparticles and carboxyl CdTe QDs, (**b**) illustration of the detection of *S*. *Typhimurium*.

The principle of the fluorescence assay for determination of *S*. *Typhimurium* is presented in Fig 2. The *S*. *Typhimurium* biotin-aptamer was bound to the surface of the streptavidin-labeled MNPs by noncovalent interactions to prepare the Apt-MNPs, and the amino-modified ssDNA2 (complementary strand of the aptamer) was labeled with carboxyl-capped CdTe QDs (QDs-ssDNA2) [20,21]. Then, the as-prepared CdTe QD-labeled ssDNA2 was incubated with the Apt-MNPs to form the aptamer-ssDNA2 duplex, which acts as a sensor detection probe. When *S*. *Typhimurium* was added, the aptamer preferentially bound to the target, which led to partial dehybridization of ssDNA2 on the surface of MNPs [22]. Then, QD-ssDNA2 was dissociated into the solution, followed by magnetic separation. Next, the supernatant was transferred into the microplate to detect and quantify the fluorescence by using a microplate reader. As a result, the fluorescence intensity change is positively related to the *S*. *Typhimurium* concentration, and determination of *S*. *Typhimurium* can be achieved.

### Demonstration of the principle

**Fig 3:**
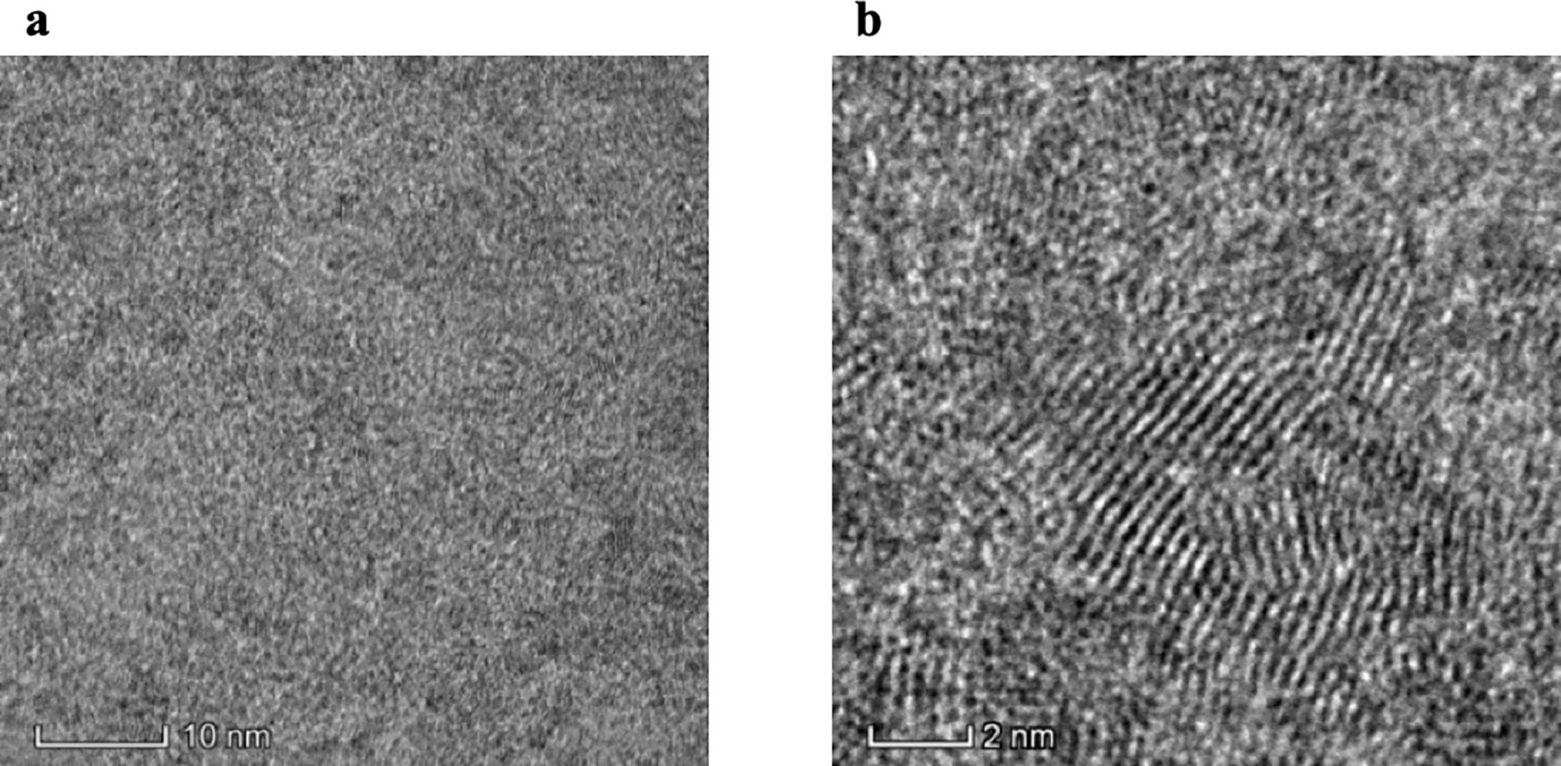
TEM (a) and HRTEM (b) images of CdTe QDs.

Fig 3 presents TEM and HRTEM images of the carboxyl-labeled CdTe QDs. The results show these QDs were nearly spherical, crystalline, monodisperse and well separated. The average size was approximately 2 nm (S1 Fig). The lattice planes were extended across the entire particles, which indicated that the CdTe QDs had a good crystallized structure.

**Fig 4:**
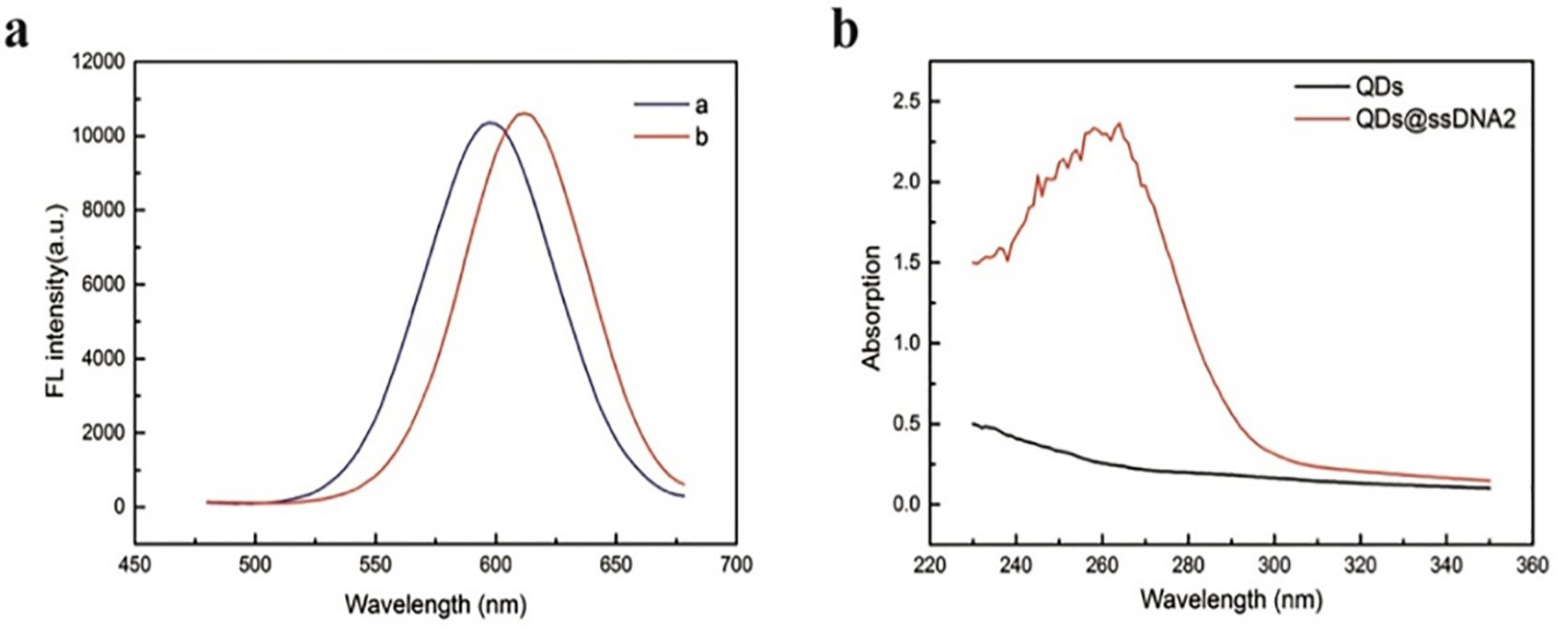
(**a**) Fluorescence spectra of QDs (curve a) and QDs@ssDNA2 (curve b); (**b**) UV-vis absorption spectrum of QDs and QDs@ssDNA2.

To confirm that ssDNA2 is modified on the surface of the CdTe QDs, different techniques were used. As shown in Fig 4A, the fluorescence spectra of the CdTe QDs showed an emission peak at 599 nm. When the CdTe QDs were labeled with ssDNA2, the emission peak exhibited a redshift from 599 nm to 612 nm. This is because the QD surface charge is altered when ssDNA2 binds to the QD surface [16,23,24]. As shown in Fig 4B, the UV-vis spectrum of the CdTe QDs shows no absorption signal; when ssDNA was added, a very high absorption signal of DNA at 260 nm appeared.

**Fig 5:**
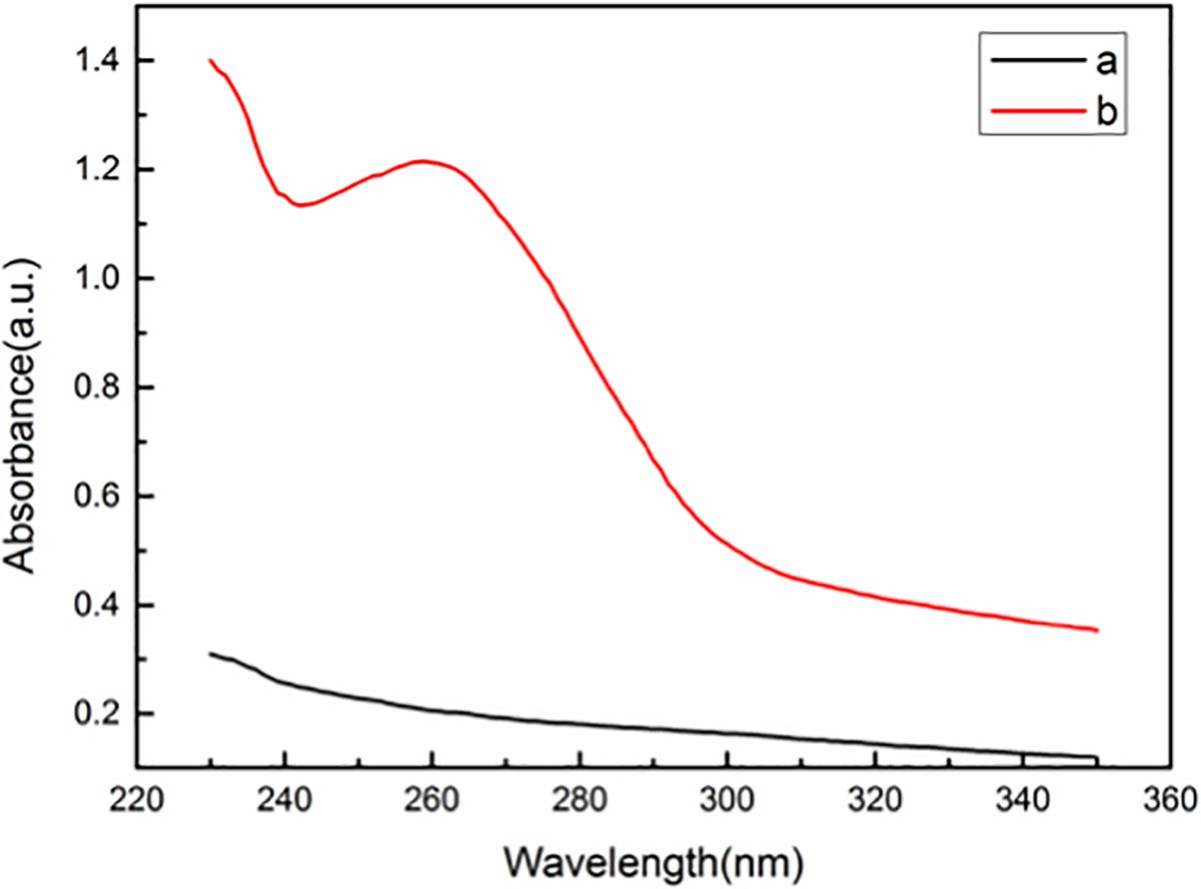
UV-vis absorption spectrum of MNPs@aptamer (curve a) and aptamer (curve b).

Next, UV-vis spectra were applied to further confirm the modification of MNPs with the aptamer hybrid. As shown in Fig 5, the aptamer DNA system showed a strong absorption peak at 260 nm (curve b), whereas no obvious absorption peak was observed for the supernatant of the MNPs/aptamer system (curve a). These results confirmed the formation of the stabilized MNPs@aptamer and its subsequent removal by magnetic separation.

### Optimization of method

**Fig 6:**
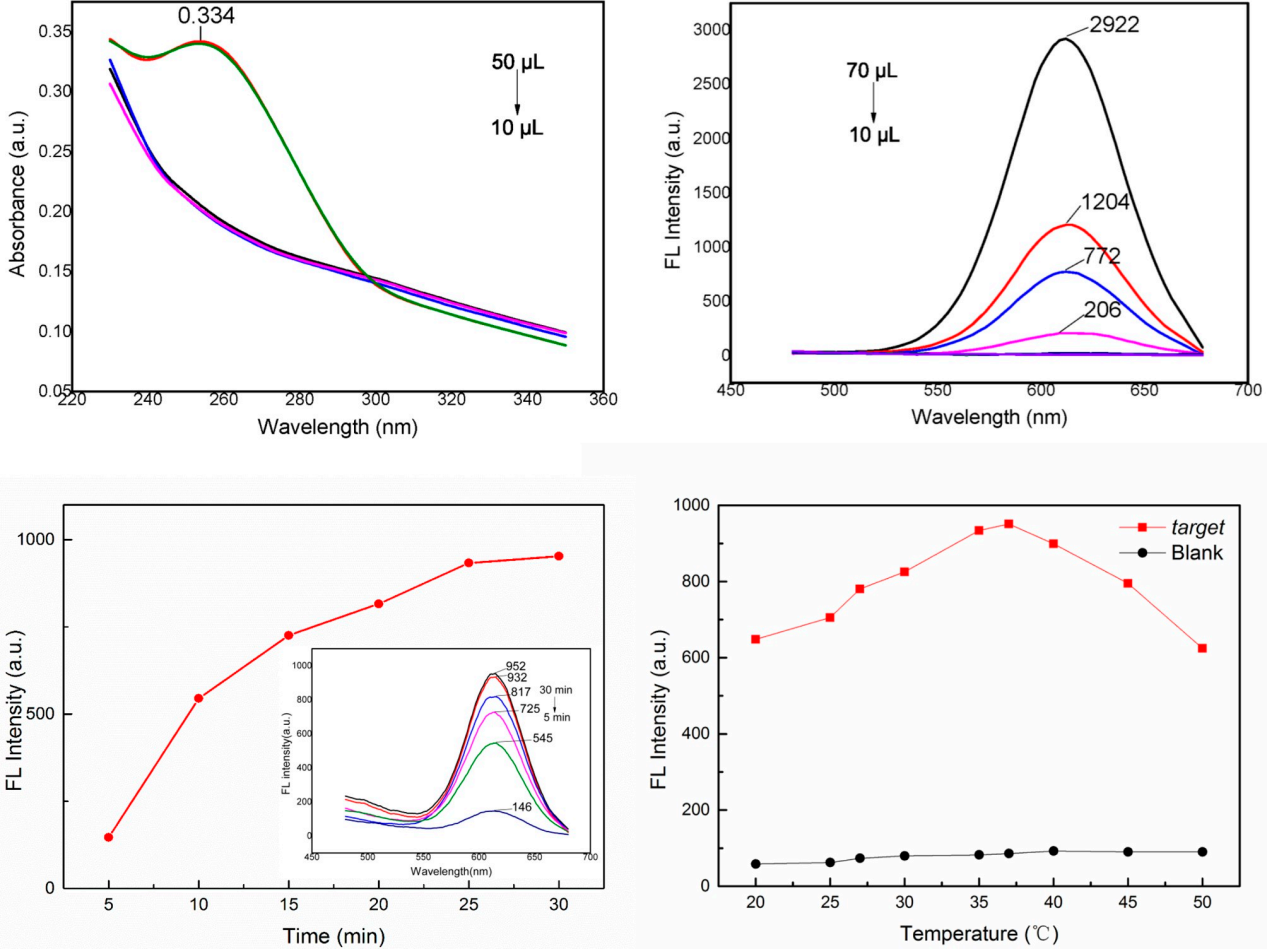
(**a**) UV-visible absorption spectrum of 10 μL of 1 mg•mL^-1^ streptavidin-coated MNPs decorated with 50 μL of 10 nM aptamer. (**b**) Fluorescence spectra of different concentrations (from 70 μL to 10 μL) of ssDNA2@CdTe QDs of 30 μg·mL^-1^ ssDNA2@CdTe QDs. (**c**) Fluorescence spectra of aptamer&QDs-ssDNA2@MNPs after different incubation times with *S*. *Typhimurium*. (**d**) Fluorescence spectra of aptamer&QDs-ssDNA2@MNPs incubated with *S*. *Typhimurium* at different incubation temperatures.

To obtain the best assay performance, some important parameters were optimized. The amount of aptamer that binds to the MNPs is a crucial parameter for sensitive detection of *S*. *Typhimurium*. A lower amount of aptamer combined with MNPs would lead to a waste of the MNPs materials and a higher limit of detection. As shown in Fig 6A, the absorption peak of aptamer-modified MNPs at 260 nm increased when the volume of biotin-labeled aptamer was 40 μL, suggesting that the saturation point was achieved under these conditions. Therefore, 10 μL of 1 mg•mL^-1^ streptavidin-coated MNPs and 40 μL of 10 nM aptamer were chosen for the following steps.

The amount of QDs-ssDNA2 is another significant factor for the detection limits because when excess QDs-ssDNA2 was added, the solution displayed high fluorescence intensity even if the target did not exist. Conversely, a low concentration of QDs-ssDNA2 can influence the sensitivity. As shown in Fig 6B, when the volume of QDs-ssDNA2 was more than 30 μL, the fluorescence intensity of CdTe QDs appeared. Conversely, when the volume of QDs-ssDNA2 was above 40 μL, the fluorescence intensity of CdTe QDs increased gradually, indicating that the optimal volume of ssDNA2@CdTe QDs is 30 μL.

Then, we confirmed the optimum incubation time between bacteria and aptamer&QD-ssDNA2@MNPs. All the samples were tested under the same conditions (37°C, 180 rpm) and bacteria concentration (10^4^ cfu•mL^-1^), but the target combination time varied (from 5 min to 30 min). Fig 6C illustrates that with prolonged incubation time, increased fluorescence intensity occurred because more ssDNA2@CdTe QDs were released. The fluorescence intensity reached a maximum and tended to be stable when the incubation time was up to 25 min. Thus, the optimal incubation time was 25 min.

We also optimized the incubation temperature of aptamer&QDs-ssDNA2@MNPs with *S*. *Typhimurium*. As shown in Fig 6D, with increasing temperature, the fluorescence intensity first increased (below 37°C) and then decreased (above 37°C), suggesting that the optimal temperature is 37°C.

Based on the results discussed above, it can be concluded that (1) the amount of aptamer that binds to the MNPs is 10 μL of 1 mg•mL^-1^ streptavidin-coated MNPs and 40 μL of 10 nM aptamer, (2) the amount of ssDNA2@CdTe QDs is 30 μL, (3) the incubation time of aptamer&QDs-ssDNA2@MNPs with bacteria is 25 min, and (4) the incubation temperature of aptamer&QDs-ssDNA2@MNPs with bacteria is 37°C.

### Fluorescent determination of *S*. *Typhimurium*

**Fig 7:**
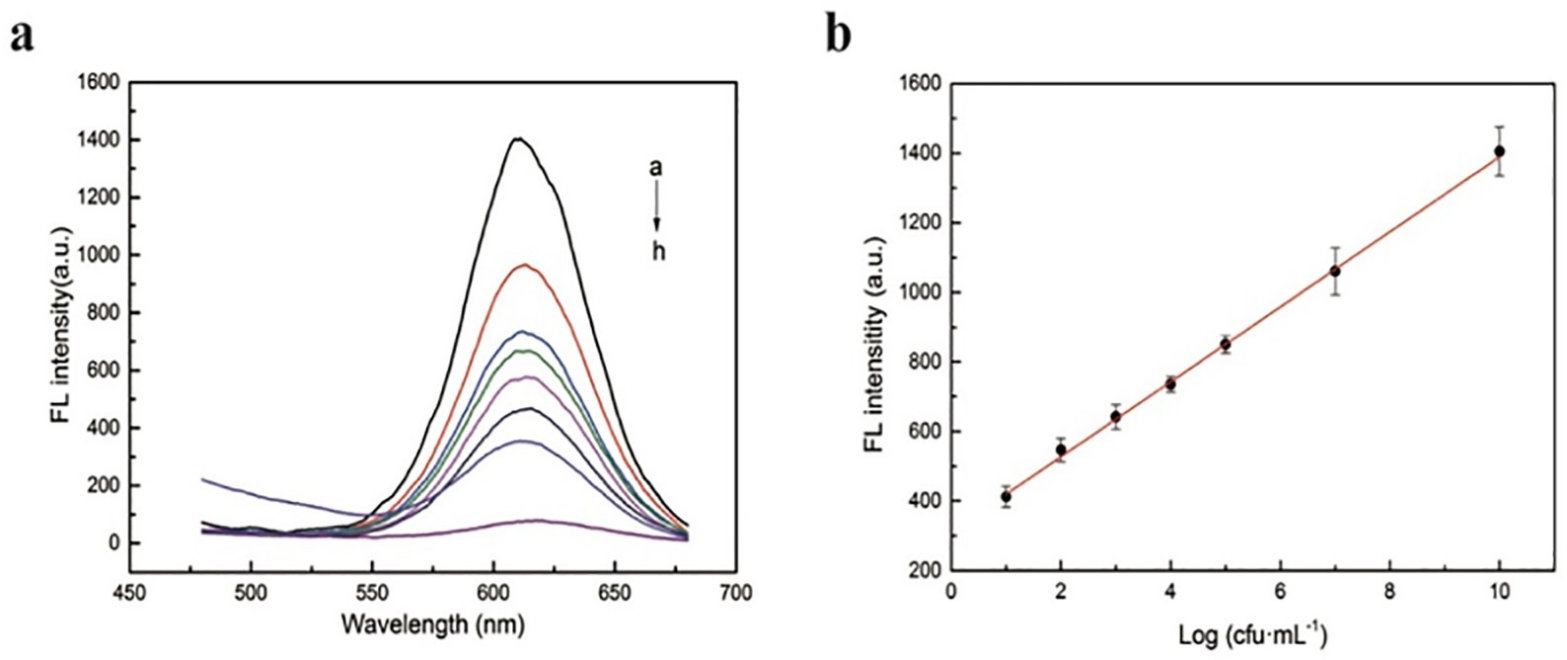
(**a**) Fluorescence spectra of aptasensors with different concentrations (from a to h: 10^10^, 10^7^, 10^5^, 10^4^, 10^3^, 10^2^, 10, 0 cfu•mL^-1^) of *S*. *Typhimurium*; (**b**) calibration curve of the fluorescence intensity of the QDs@ssDNA2 at 612 nm for *S*. *Typhimurium* detection.

Under optimized conditions, various amounts of *S*. *Typhimurium* were added to suspensions of aptamer& QDs-ssDNA2@MNPs to verify the sensitivity of the assay (Fig 7). The emission peak shifts from approximately 599 to 612 nm when ssDNA2 binds to the QD surface. Therefore, the fluorescence intensity at 612 nm was measured. The results showed that there is an obvious increase in the fluorescence intensity with increased *S*. *Typhimurium* concentration (Fig 7A). This is caused by more dissociation of QD-ssDNA2 conjugates on the MNP surface.

Fig 7B reveals a linear relationship between fluorescence intensity and the logarithm of *S*. *Typhimurium* concentration from 10^1^ cfu•mL^-1^ to 10^10^ cfu•mL^-1^, with R^2^ = 0.99. The regression equation of the calibration curve was *y = 107*.*74x+312*.*15*. The detection limit of *S*. *Typhimurium* is calculated to be 1 cfu•mL^-1^. The results indicated that the assay can be employed to detect and quantify *S*. *Typhimurium* with strong sensitivity and a wide range of concentrations. The comparison between this method and other approaches to detect *S*. *Typhimurium* is summarized in Table 1. The assay has distinctive advantages, such as wide dynamic range, good detection limit, simplicity, rapidness, and low cost. Most of all, these results indicated that the fluorescence method can be applied to the detection of *S*. *Typhimurium* with great potential.

**Table 1:** Comparison of different methods for the detection of *S*. *Typhimurium*.

Methods	Linear range (cfu·mL^-1^)	LOD (cfu·mL^-1^)	Recognition element	Reference
DNA-assembled gold nanodimers	10^2^−10^7^	35	Aptamer	[25]
Microfluidic nanobiosensor	10^3^−10^6^	10^3^	Polyclonal antibodies	[26]
Graphene oxide platform	10^3^−10^8^	100	Aptamer	[27]
F0F1-ATPase biosensor	10^1^−10^4^	10	Aptamer	[28]
IMS-PMA-mPCR	10^1^−10^7^	10	Polyclonal antibody	[29]
Immunomagnetic nanoparticle-based quantitative PCR	10^3^−10^5^	10^3^	Antibody	[30]
ELISA	10^3^−10^8^	10^3^	Gold nanoparticle-based enzyme-linked antibody-aptamer sandwich	[31]
Surface-enhanced Raman scattering	10^3^−10^6^	10^3^	Antibody	[32]
Digital PCR	2.5×10^2^−2.5×10^6^	2.5×10^2^	Specific nucleic acid	[33]
Aptamer modified magnetic nanoparticles	10−10^10^	1	Aptamer	This study

### Selectivity and interference of the assay

**Fig 8:**
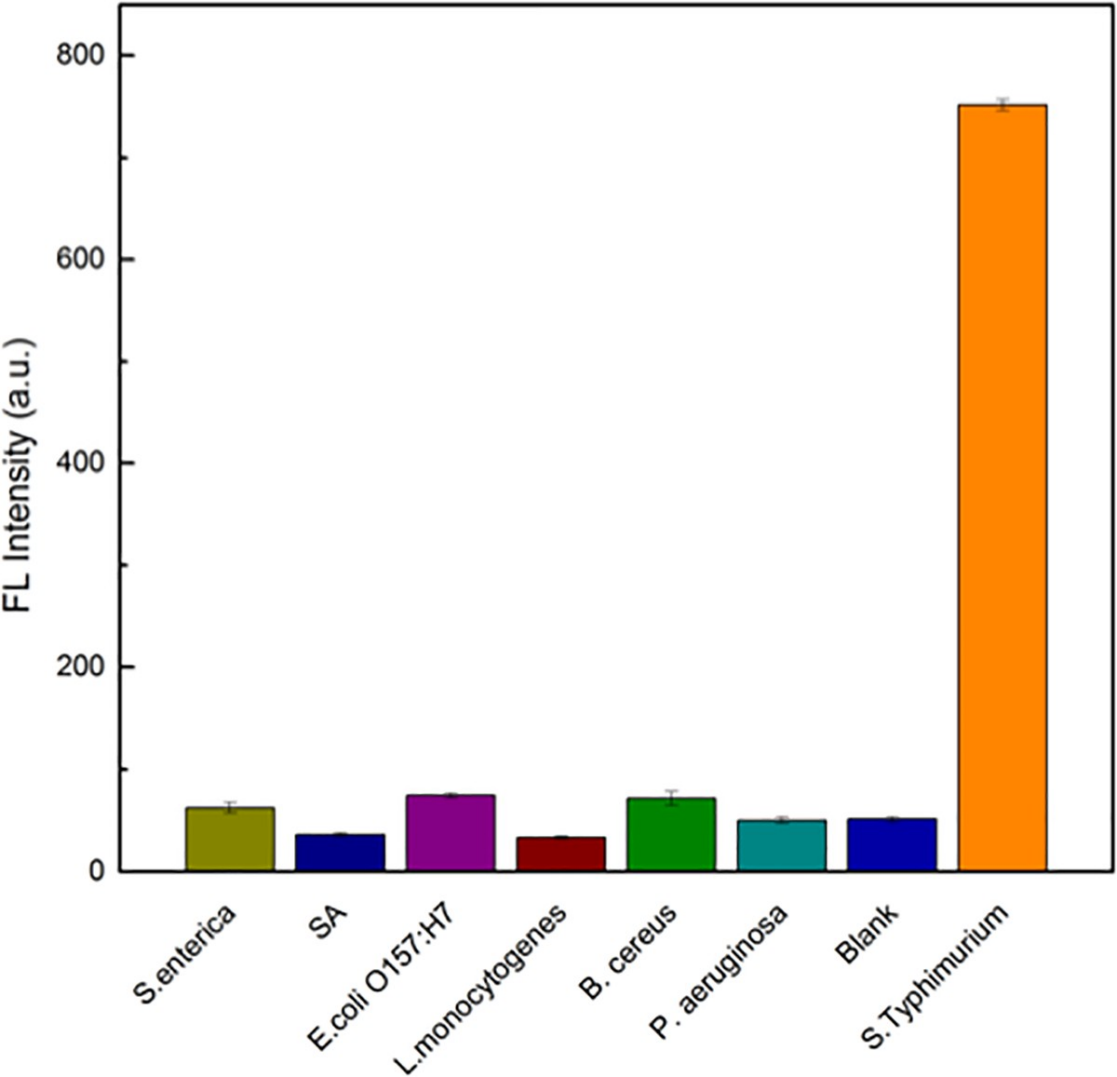
Specificity result for the detection of *S*. *enteritidis*, *S*. *aureus*, *E*. *coli O157*:*H7*, *L*. *monocytogenes*, *B*. *cereus*, *P*. *aeruginosa* and *S*. *Typhimurium*.

To evaluate the specificity of the assay, other foodborne pathogens, including *Staphylococcus aureus*, *Escherichia coli* O157:H7, *Listeria monocytogenes*, *Bacillus cereus*, *Salmonella enteritidis* and *Pseudomonas aeruginosa* as negative controls, were added to the system. The concentration of *S*. *Typhimurium* and other pathogens was 10^4^ cfu•mL^-1^, and the fluorescence intensity after incubation with various pathogens for 25 min at 37°C was calculated. As shown in Fig 8, only *S*. *Typhimurium* can induce a remarkable increase in fluorescence intensity. This is because of the high affinity between the aptamer and *S*. *Typhimurium*, while other pathogens have weak binding with the aptamer of *S*. *Typhimurium*. The results suggest that the assay we established has good specificity and selectivity towards *S*. *Typhimurium*.

### Real sample detection

Drinking water and milk were used as real samples to validate the applicability and feasibility of this assay. First, no *S*. *Typhimurium* was detected in the samples by the plate counting method. Then, different amounts of *S*. *Typhimurium* were added to drinking water and milk and then detected by using the aptamer& QDs-ssDNA2@MNPs assay and plate counting method. As depicted in Table 2, the recoveries of the spiked milk and water samples ranged from 95% to 110% with good analytical precision (RSD<10%), which is in agreement with data obtained from the plate counting method. The results indicated that the method has a potential application to detect *S*. *Typhimurium* in real samples.

**Table 2:** Determination of *S*. *Typhimurium* in real samples.

Sample	Plate counting (cfu·mL^-1^)	This method		
		Found (cfu·mL^-1^)	Recovery (%)	RSD (%)
Milk 1	(2.48±0.21) ×10	(2.51±0.14) ×10	101.2	5.6
Milk 2	(6.76±0.49) ×10^3^	(6.47±0.26) ×10^3^	95.7	4.1
Milk 3	(7.35±0.55) ×10^4^	(7.58±0.49) ×10^4^	103.1	6.5
Water 1	(5.27±0.30) ×10	(5.09±0.38) ×10	96.6	7.5
Water 2	(4.48±0.56) ×10^3^	(4.61±0.42) ×10^3^	102.9	9.7
Water 3	(5.81±0.52) ×10^4^	(5.64±0.27) ×10^4^	97.1	4.8

## Conclusions

In this paper, we report a magnetic separation system-based fluorescence sensing strategy to detect and quantify *S*. *Typhimurium* with high sensitivity and selectivity. QD-ssDNA2 was incubated with Apt-MNPs to form an aptamer-complementary DNA duplex as a detection probe. Upon addition of *S*. *Typhimurium*, QD-ssDNA2 is replaced by the bacteria and released from the Fe_3_O_4_ MNPs, accompanied by the release of CdTe QD-labeled ssDNA2, resulting in a significantly increased fluorescence intensity. The difference in fluorescence intensity can be used to sensitively detect *S*. *Typhimurium*, with a low detection limit of 1 cfu•mL^-1^. Furthermore, the fluorescence sensor was also successfully applied to detect *S*. *Typhimurium* in water and milk. In addition, the aptamer and QD-modified complementary sequences are more cost-effective and more stable than antibodies, and the assay can capture targets with magnetic beads and use fluorescence to quantify *S*. *Typhimurium* simultaneously within 2 h. In conclusion, the sensing assay has the potential to be further extended to on-site screening of pathogenic bacteria *S*. *Typhimurium-*related food contamination and other pathogenic bacteria targets by changing the aptamer and cDNA.

## Supporting information

S1 Fig The DLS result of CdTe QDs. (DOCX)
