# Supplementary figures and images for: Aptamer-based fluorometric determination of Salmonella Typhimurium using Fe3O4 magnetic separation and CdTe quantum dots
Source: PLoS One. 2019 Jun 19;14(6):e0218325. doi: 10.1371/journal.pone.0218325 (PMC6584018; doi:10.1371/journal.pone.0218325)

**Supporting information**

Fig S1. The DLS result of CdTe QDs.


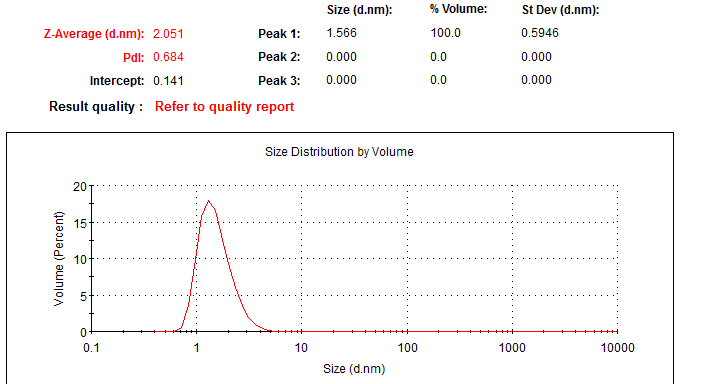

Supplement: S1 Fig — (DOCX) [file pone.0218325.s001.docx]
